# Comparison of Care Cascade Outcome Measures for Hepatitis C Among Insured US Adults

**DOI:** 10.1001/jamanetworkopen.2026.21736

**Published:** 2026-07-06

**Authors:** Hasan Symum, Brooke Hoots, Harvey W. Kaufman, William A. Meyer, Ademola Osinubi, Andrew Kress, William W. Thompson, Carolyn Wester

**Affiliations:** 1 Division of Viral Hepatitis, National Center for HIV, Viral Hepatitis, STD, and Tuberculosis Prevention, Centers for Disease Control and Prevention, Atlanta, Georgia; 2 Department of Pediatrics, Boston Children’s Hospital, Boston, Massachusetts; 3 Quest Diagnostics, Secaucus, New Jersey; 4 HealthVerity, Philadelphia, Pennsylvania

## Abstract

This cohort study compares hepatitis C care cascade outcome measures using laboratory and pharmacy data to document viral clearance and treatment initiation, respectively, among insured US adults.

## Introduction

Untreated hepatitis C virus (HCV) can lead to advanced liver disease, cancer, and death, although direct-acting antivirals (DAAs) cure most (>95%) cases.^1^ Sustained virologic response (SVR; evidenced by undetectable HCV RNA≥12 weeks after treatment completion) indicates cure.^2^ Different data sources have been used to monitor successful hepatitis C treatment,^3^ including laboratory data to document viral clearance, pharmacy data to document treatment initiation, or both.^4,5^ Health care systems often have access to only 1 data source; therefore, comparing outcomes is essential for measuring progress toward hepatitis C elimination.

## Methods

This cohort study was reviewed by the Centers for Disease Control and Prevention (CDC), deemed not research, and conducted consistent with applicable federal law and CDC policy. We followed the STROBE guideline.

The HealthVerity database includes longitudinal laboratory results, pharmacy claims, and insurance enrollment information (eMethods in Supplement 1). Eligible US adults (aged ≥18 years) who underwent HCV antibody, RNA, or genotype testing between January 30, 2019, and June 30, 2022, were identified. Those who ever had HCV infection were included if they had positive or detectable HCV (between January 30, 2019, and June 30, 2021), continuous insurance enrollment (≥60 days before and 360 days after the index date [earliest date of any positive HCV result]), and no evidence of DAA treatment 60 days before the index date.

We calculated the initial steps of a hepatitis C care cascade (ever had HCV infection, viral testing, and initial infection) using laboratory results as described.^3^ Three care outcomes were calculated overall and by demographics: viral clearance (HCV RNA-negative or undetectable results among those with initial infection), treatment initiation (DAA prescription ≤360 days of initial infection), and viral clearance after treatment initiation (used as a proxy for SVR; negative or undetectable HCV RNA result after treatment initiation). Outcomes were not mutually exclusive. Data were analyzed between March 2024 and January 2025.

## Results

Of 4 515 568 adults with at least 1 HCV test, 91 491 (mean [SD] age, XX [XX] years; 46.8% female, 53.2% male) ever had HCV (Table 1). Most (82 433 [90.1%]) underwent at least 1 HCV RNA test. Nearly two-thirds (51 068 [62.0%]) had initial HCV infection; 39.9% had viral clearance, 35.7% initiated treatment, and 26.1% had viral clearance after treatment initiation. Similar trends were observed in care outcomes across age groups and payers, with different magnitudes.

**Table 1.** HCV Infection Outcome Measures, by Demographic Characteristics (January 30, 2019, to June 30, 2022)

Characteristic	No. of persons who ever had HCV infection (N = 91 491)^c^	No. (%) of persons^a^				
		Viral testing (n = 82 433 [90.1%])^c^	Initial infection (n = 51 068 [62.0%])	Outcomes among persons with initial infection^b^		
				Viral clearance (n = 20 390 [39.9%])	Treatment initiation (n = 18 246 [35.7%])	Viral clearance after treatment initiation (n = 13 351 [26.1%])
Age group, y						
18-29	12 367	11 037 (89.2)	7342 (66.5)	2448 (33.3)	1974 (26.9)	1309 (17.8)
30-39	20 316	18 206 (89.6)	12 615 (69.3)	4637 (36.8)	4283 (34.0)	2985 (23.7)
40-49	15 183	13 747 (90.5)	8945 (65.1)	3574 (40.0)	3359 (37.6)	2388 (26.7)
50-59	24 300	22 022 (90.6)	13 006 (59.1)	5641 (43.4)	5163 (39.7)	3952 (30.4)
≥60	19 325	17 421 (90.1)	9160 (52.6)	4090 (44.7)	3467 (37.8)	2717 (29.7)
Sex						
Female	42 802	38 411 (89.7)	22 118 (57.6)	8871 (40.1)	7704 (34.8)	5627 (25.4)
Male	48 689	44 022 (90.4)	28 950 (65.8)	11 519 (39.8)	10 542 (36.4)	7724 (26.7)
Payer						
Commercial	13 388	12 151 (90.8)	5700 (46.9)	3166 (55.5)	2772 (48.6)	2174 (38.1)
Medicare	8399	7544 (89.8)	3678 (48.8)	1565 (42.6)	1470 (40.0)	1115 (30.3)
Medicaid	69 704	62 738 (90.0)	41 690 (66.5)	15 659 (37.6)	14 004 (33.6)	10 062 (24.1)

Abbreviations: DAA, direct-acting antiviral; HCV, hepatitis C virus.

^a^ Denotes the conditional proportion (viral testing and initial infection) based on the preceding column. Row percentages are reported, which do not sum to 100%.

^b^ Assessed among persons with initial infection as follows: viral clearance (≥1 undetectable HCV RNA test result after a previous detectable result, irrespective of treatment status), treatment initiation (dispensing of at least 1 DAA prescription after initial infection), and viral clearance after treatment initiation (undetectable HCV RNA result occurring after DAA treatment began). The conditional proportion represents the proportion of persons with initial HCV infection who had evidence of viral clearance, treatment initiation, and viral clearance after treatment initiation.

^c^ Defined based on HCV laboratory results as follows: ever had HCV infection (persons with any detectable HCV antibody, RNA, or genotype results), viral testing (receipt of any HCV RNA test result [detectable or undetectable] after a positive HCV test), and initial infection (first occurrence of a detectable HCV RNA result, representing active HCV infection).

Laboratory result–based care outcomes were limited by follow-up HCV RNA testing (Table 2). Among persons with initial infection, 19 545 (38.3%) did not have a subsequent RNA test to assess viral clearance; 78.4% had no evidence of treatment initiation. Among 18 246 individuals who initiated treatment, 73.2% had a subsequent HCV RNA-negative result, 3.7% had an RNA-positive result, and 23.2% had no evidence of subsequent testing. Most of the latter (3840 of 4229 [90.8%]) were prescribed at least 56 days of DAA treatment. Among 32 822 individuals without treatment initiation, 21.3% subsequently had an RNA-negative result, 32.1% had an RNA-positive result, and 46.7% had no subsequent testing.

**Table 2.** HCV RNA Test Results Among Persons With Initial Infection, by Treatment Initiation

Treatment initiation^b^	No. of persons with initial HCV infection (n = 51 068)^c^	HCV RNA testing after initial infection^a^		
		≥1 Negative result (cured or cleared) (n = 20 327 [39.8%])	Positive result only (not cured or cleared) (n = 11 196 [21.9%])	No test (unknown if cured or cleared) (n = 19 545 [38.3%])
HCV DAA treatment supply, d	18 246	13 351 (73.2)	666 (3.7)	4229 (23.2)
<56	1039	495 (47.6)	155 (14.9)	389 (37.4)
≥56	17 207	12 856 (74.7)	511 (3.0)	3840 (22.3)
Not treated	32 822	6976 (21.3)	10 530 (32.1)	15 316 (46.7)

Abbreviations: DAA, direct-acting antiviral; HCV, hepatitis C virus.

^a^ Values are reported as No. (%) of persons. Row percentages are reported, which do not sum to 100%.

^b^ Treatment initiation was defined as at least 1 prescription for DAAs within 360 days after initial infection.

^c^ Initial infection was defined as the first occurrence of a detectable HCV RNA result, representing active HCV infection.

## Discussion

Although viral clearance requires only laboratory data, actual clearance can be underestimated owing to lack of follow-up HCV testing or overestimated due to inclusion of self-limited infection.^6^ Here, 23.2% of individuals who initiated treatment did not have subsequent test results, yet 91.0% were prescribed DAAs (≥56 days; eg, two 28-day prescriptions) and likely achieved treatment-induced viral clearance.^7^ Conversely, 21.3% of individuals without treatment initiation had evidence of viral clearance, suggesting spontaneous clearance or treatment not captured in HealthVerity data.^8^

Hepatitis C care outcomes based on pharmacy data (alone or with laboratory data) allow for assessment of treatment initiation. For databases without laboratory data, our results suggest pharmacy data are a reasonable proxy for cure due to the high cure percentage of DAAs.^3^ However, treatment initiation based on pharmacy data alone may overestimate the proportion cured, owing to nonadherence.^9^

Viral clearance after treatment initiation, which requires both laboratory and pharmacy data, is the most rigorous outcome and most consistent with the definition of SVR.^2,10^ However, it likely underestimates cure because many patients who initiate treatment do not return for follow-up RNA testing.

This study has limitations. Treatments occurring outside of insurance claims may not have been captured. Evolving CDC and US Preventive Services Task Force recommendations may have influenced testing patterns and cohort entry. The requirement for continuous enrollment may exclude individuals with unstable insurance coverage. Additionally, misclassification of infection status or outcomes is possible due to reliance on laboratory data availability.

Measuring care outcomes is essential to monitoring progress toward hepatitis C elimination. Our findings suggest that improving follow-up RNA testing and integrating laboratory and pharmacy data will enhance accuracy and bridge gaps in hepatitis C testing and treatment. However, when only one data source is available, viral clearance or treatment initiation can serve as reasonable substitutes for measuring viral clearance after treatment initiation.

## Notes

Supplement 1. **eFigure.** Cohort Selection for Patients With Initial Hepatitis C Infection
eMethods

Supplement 2.
Data Sharing Statement
