# Supplementary material for: Comparison of Care Cascade Outcome Measures for Hepatitis C Among Insured US Adults
Source: JAMA Netw Open. 2026 Jul 6;9(7):e2621736. doi: 10.1001/jamanetworkopen.2026.21736 (PMC13338805; doi:10.1001/jamanetworkopen.2026.21736)
Supplement: Supplement 1. — eFigure. Cohort Selection for Patients With Initial Hepatitis C Infection eMethods [file jamanetwopen-e2621736-s001.pdf]

Supplementary Online Content

Symum H, Hoots B, Kaufman HW, et al. Comparison of care cascade outcome measures for hepatitis C among insured US adults. *JAMA Netw Open*. 2026;9(7):e2621736. doi:10.1001/jamanetworkopen.2026.21736

eFigure. Cohort Selection for Patients With Initial Hepatitis C Infection

eMethods

This supplementary material has been provided by the authors to give readers additional information about their work.

© 2026 Symum H et al. *JAMA Network Open*.

eFigure. Cohort Selection for Patients With Initial Hepatitis C Infection

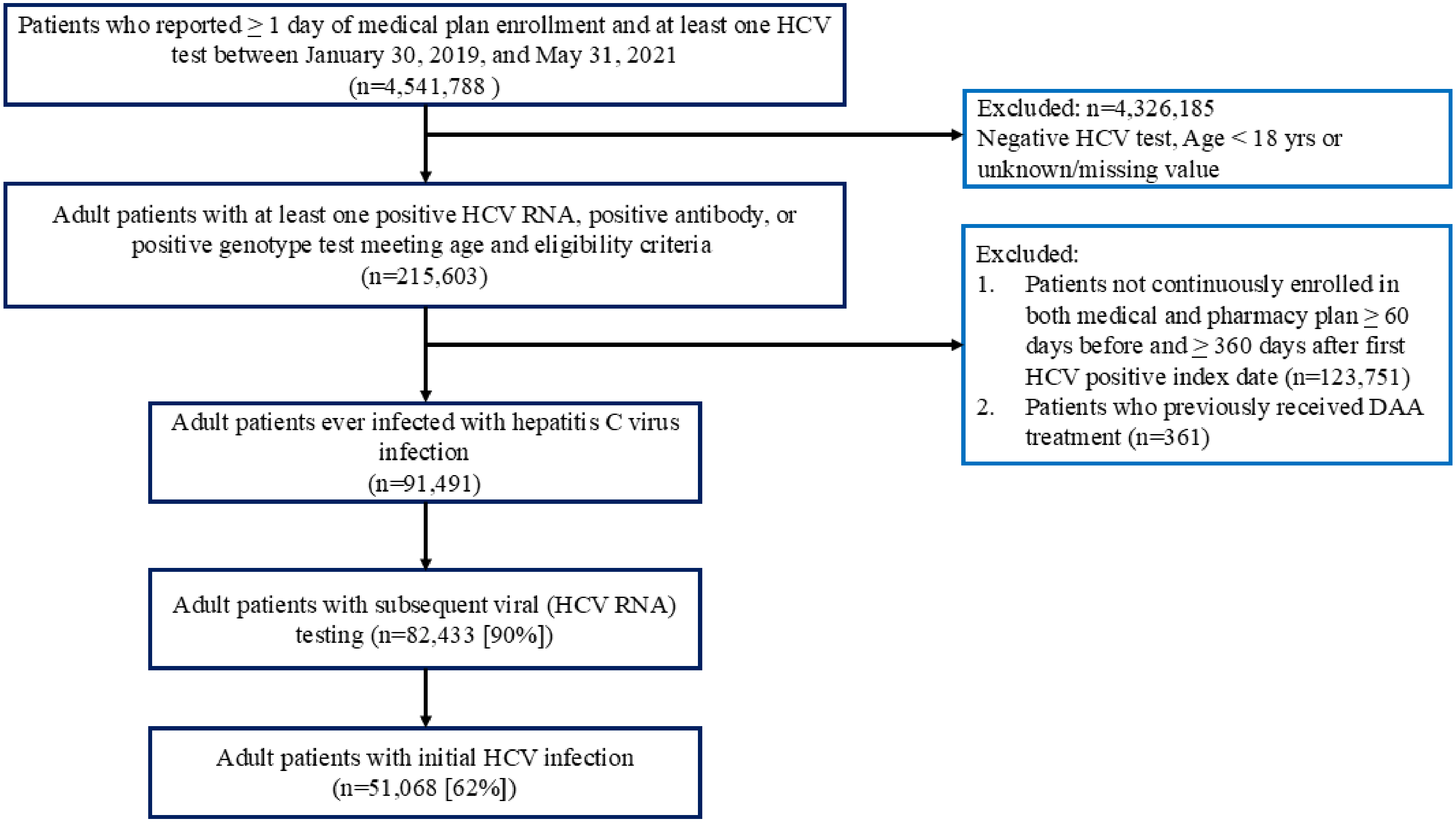

© 2026 Symum H et al. *JAMA Network Open*.

eMethods

Dataset: HealthVerity is a large, de-identified, longitudinal real-world data platform that links administrative claims (medical and pharmacy) with laboratory test results across multiple payer types, including Medicaid, Medicare, and commercial insurance, covering all 50 U.S. states. Data are integrated from numerous sources within the HealthVerity Marketplace (HealthVerity Marketplace: Access Real-World Healthcare Data), one of the largest U.S. healthcare data ecosystems, encompassing over 340 million de-identified individuals and multiple data types, including claims, pharmacy, and laboratory data. Laboratory results are derived from national reference laboratories and are linkable to claims at the patient level through privacy-preserving tokens, enabling longitudinal tracking across care settings. HCV testing was identified using linked laboratory results based on Logical Observation Identifiers Names and Codes (LOINC), which are standardized within the HealthVerity data model. Limitations: We acknowledge several limitations. Because we used closed claims data, direct-acting antiviral (DAA) prescriptions reimbursed through insurance, including those dispensed through specialty pharmacies, should be captured; however, treatments paid out-of-pocket, obtained through patient assistance programs, or otherwise occurring outside of insurance claims may not be observed. Evolving Centers for Disease Control and Prevention and U.S. Preventive Services Task Force screening recommendations during the study period may have influenced testing patterns and cohort entry. The requirement for continuous enrollment may exclude individuals with unstable insurance coverage, including populations at higher risk for HCV infection (e.g., people who inject drugs), although it improves completeness of captured pharmacy and laboratory data for assessing treatment initiation and follow-up. Additionally, misclassification of infection status or outcomes is possible due to reliance on laboratory data availability (LOINC-coded results). These considerations should be taken into account when interpreting the findings.

© 2026 Symum H et al. *JAMA Network Open*.
