# Supplementary material for: Comparison of Care Cascade Outcome Measures for Hepatitis C Among Insured US Adults
Source: JAMA Netw Open. 2026 Jul 6;9(7):e2621736. doi: 10.1001/jamanetworkopen.2026.21736 (PMC13338805; doi:10.1001/jamanetworkopen.2026.21736)
Supplement: Supplement 2. — Data Sharing Statement [file jamanetwopen-e2621736-s002.pdf]

Data Sharing Statement

Symum. Comparison of Care Cascade Outcome Measures for Hepatitis C Among Insured US Adults. *JAMA Netw Open*. Published July 06, 2026. doi:10.1001/jamanetworkopen.2026.21736

Data
Data available: No

Additional Information
Explanation for why data not available: The data used in this study are a proprietary dataset provided by HealthVerity and are subject to data use agreements. Therefore, datasets are not publicly available.
